# The early impacts of primary HPV cervical screening implementation in Australia on the pathology sector: a qualitative study

**DOI:** 10.1186/s12913-023-10040-6

**Published:** 2023-10-06

**Authors:** Claire Bavor, Julia ML Brotherton, Megan A Smith, Khic-Houy Prang, Tracey McDermott, Nicole M Rankin, Claire M Zammit, Chloe J Jennett, Farhana Sultana, Dorothy A Machalek, Claire E Nightingale, on behalf of the STORIES team

**Affiliations:** 1 Centre for Health Policy, Melbourne School of Population and Global Health, University of Melbourne, Melbourne, VIC 3010 Australia; 2 Formerly employed by the Australian Centre for the Prevention of Cervical Cancer, Carlton, VIC 3053 Australia; 3 The Daffodil Centre, the University of Sydney, a joint venture with Cancer Council NSW and the University of Sydney, Sydney, NSW 2011 Australia; 4 National Cancer Screening Register, Telstra Health, Melbourne, VIC 3000 Australia; 5 The Kirby Institute, University of New South Wales, Kensington, Sydney, NSW 2052 Australia; 6 Centre for Women’s Infectious Diseases, The Royal Women’s Hospital, Parkville, VIC 3052 Australia

**Keywords:** Cervical screening, Cervical cancer, Pathology, HPV, Implementation, Qualitative, Self-collection, Change management, Self-sampling

## Abstract

**Background:**

The transition of Australia’s National Cervical Screening Program from cytology to a molecular test for human papillomavirus (HPV) (locally referred to as the ‘Renewal’), including a longer five-year interval and older age at commencement, significantly impacted all sectors of program delivery. The Renewal had major implications for the roles and requirements of pathology laboratories providing services for the Program. This study aimed to understand the early impacts of the Renewal and its implementation on the pathology sector.

**Methods:**

Semi-structured qualitative interviews were conducted with key stakeholders (N = 49) involved in the STakeholder Opinions of Renewal Implementation and Experiences Study (STORIES), 11–20 months after the program transition. A subset of interviews (N = 24) that discussed the pathology sector were analysed using inductive thematic analysis.

**Results:**

Four overarching themes were identified: implementation enablers, challenges, missed opportunities, and possible improvements. Participants believed that the decision to transition to primary HPV screening was highly acceptable and evidence-based, but faced challenges due to impacts on laboratory infrastructure, resources, staffing, and finances. These challenges were compounded by unfamiliarity with new information technology (IT) systems and the new National Cancer Screening Register (‘Register’) not being fully functional by the date of the program transition. The limited availability of self-collection and lack of standardised fields in pathology forms were identified as missed opportunities to improve equity in the Program. To improve implementation processes, participants suggested increased pathology sector involvement in planning was needed, along with more timely and transparent communication from the Government, and clearer clinical management guidelines.

**Conclusion:**

The transition to primary HPV screening had a significant and multifaceted impact on the Australian pathology sector reflecting the magnitude and complexity of the Renewal. Strategies to support the pathology sector through effective change management, clear, timely, and transparent communication, as well as adequate funding sources will be critical for other countries planning to transition cervical screening programs.

**Supplementary Information:**

The online version contains supplementary material available at 10.1186/s12913-023-10040-6.

## Key message

- The transition of Australia’s National Cervical Screening Program from cytology to a molecular test for primary HPV screening had a significant and multifaceted impact on the pathology sector’s role and responsibilities in program delivery.
- Key implementation challenges experienced by laboratories were linked to managing complex workflows, increased workloads, staffing changes, new IT systems and infrastructure, and increased expenditure.
- To support the implementation of primary HPV screening by laboratories, program and policy stakeholders should facilitate effective change management, early and active engagement with the pathology sector, adequate funding, and clear and timely communication.

## Background

Australia’s National Cervical Screening Program (‘Program’) was established in 1991 and recommended two-yearly conventional Papanicolaou (Pap) testing [1]. While this Program was successful in halving cervical cancer incidence, [2] emerging evidence of the superiority of primary human papillomavirus (HPV) screening over Pap testing, [3, 4] alongside the introduction of prophylactic HPV vaccination, led to a major review of the Program in 2017 (the ‘Renewal’). Primary HPV screening was introduced with triage based on partial genotyping and liquid-based cytology (LBC). Unlike cervical cytology, which looks for precancerous (or potentially cancerous) changes in a sample of cells from the cervix, primary HPV screening uses clinically calibrated molecular tests to determine whether oncogenic HPV, the primary cause of these changes, is present at levels associated with the presence of a precancerous lesion [1]. The fundamental differences in these screening technologies have significant implications for the role, activities, and resource requirements of the pathology sector in a cervical screening program, especially for settings like Australia with an established and successful cytology-based program.

Prior to the Renewal, resource and workforce challenges were predicted for the pathology sector as the recommended screening interval increased from two years to five causing fluctuations in screening participation in the first screening rounds [5]. Other workforce challenges were considered and included the impact of the reduced need for cervical cytology on the cytology workforce size and skills, as well as the need to maintain quality and safety [6, 7]. As a result, a Renewal Steering Committee prioritised the development of quality measures and standards for HPV testing and LBC [8]. New evidence-based criteria were implemented under a quality-based requirements framework to allow for laboratories to select the best HPV nucleic acid testing (NAT) assay for their needs under the performance standards and characteristics required by Australia’s Pathology Accreditation Advisory Council [9].

Another key Program change included a new National Cancer Screening Register (‘Register’) of participants’ screening history to support pathologists in making appropriate recommendations for clinical management [10]. The Register consolidated eight jurisdictional Pap smear registers and replaced a system that only sent reminders to participants once they had been screened at least once and were overdue with a call/recall system to actively send invitations and reminders. The date of the Renewal was anticipated to start from May 1 2017, with laboratories, in preparation for the transition to large-scale HPV testing, [5] significantly reducing their cytology workforce. However, the development and launch of the Register was substantially delayed, pushing the start date to December 1 2017 [11]. This delay led to a financial subsidy from the Government to laboratories to enable them to maintain services in accordance with the original Program [11].

The implementation of the Renewal was coordinated by the Australian Government, with State and Territory jurisdictions having local oversight of program delivery and management [8]. The Program is primarily delivered through primary care and funded publicly through Medicare, Australia’s universal healthcare system; however, screening participants may be charged a gap fee by primary care practitioners for a consultation or by pathology laboratories [8]. Pathology services are provided by public and private laboratories that meet the requirements for reporting tests for the Program [12].

We undertook a series of interviews with key program stakeholders, in the STakeholder Opinions of Renewal Implementation and Experiences Study (STORIES), to understand the early impacts of the Renewal and its implementation. The key implementation challenges across several Program stakeholder groups have been described previously [13, 14]. This study aimed to understand the early impacts of Renewal and its implementation on the pathology sector.

## Methods

### Study design

This qualitative study consisted of semi-structured interviews conducted with key stakeholders directly and indirectly involved in the implementation of the Renewal [13, 14]. Key stakeholders were interviewed about their perspectives and experiences of being involved in the implementation of the Renewal, including the associated barriers, challenges, and enablers.

### Recruitment

Purposive sampling was used to recruit potential participants. Participants were eligible if they were directly or indirectly involved in the implementation of the Renewal in Australia. This included cervical screening program and policy staff, cervical screening providers, and the pathology sector. Potential participants were identified through the networks of the STORIES team and the study Advisory Committee and invited to provide perspectives from a variety of roles, areas of expertise, and locations. Participants were emailed an invitation letter and plain language statement outlining the study and their role in it.

### Materials

An interview guide was developed by the study team after a workshop with the STORIES investigators to systematically identify the possible impacts of the Renewal on key stakeholders. Questions were then developed and aligned to Proctor’s Conceptual Framework for Implementation Outcomes [15]. The eight outcomes include acceptability, adoption, appropriateness, feasibility, fidelity, implementation cost, penetration and sustainability [15]. The interview guide was tailored for each stakeholder group in consultation with the study Advisory Committee. The interview guide has been published elsewhere [14].

### Data collection

Interviews were conducted online, by phone or face-to-face by several members of the research team all of whom were trained in qualitative interviewing (JB, MS, TM, NR, FS, DM). Interviews were audio-recorded and transcribed verbatim. Transcripts were sent to the study participants for review of their accuracy and completeness if requested. Study participants were asked about the acceptability of the Renewal for themselves or their organisation, and other stakeholders; the impact of the Renewal and challenges in implementing it; and how this could have been improved. While the prompts in the interview guide were tailored to each stakeholder group, there were no specific questions about the impact of the Renewal on the pathology sector. This meant that STORIES participants across non-pathology stakeholder groups raised issues relating to the pathology sector only where they thought it was relevant.

### Data analysis

Inductive thematic analysis was conducted to identify, analyse and report themes in the data [16]. Two authors (KP and TM) reviewed a sample of five STORIES transcripts to independently develop coding trees. These were discussed and compared to reach an agreed coding tree to organise the overarching themes. One author (TM) coded the remaining transcripts and new codes were added if required [15]. For this study, all the data that had been coded as being related to the pathology sector underwent a second round of coding using inductive thematic analysis by one researcher (CB), a research assistant with formal university level training in qualitative research, to identify the themes specific to the impact of the Renewal on the pathology sector. The team reviewed and discussed these themes iteratively. NVivo 11 and NVivo 12 were used to analyse the data. The data reported in this paper focuses on the impact of the renewal on the pathology sector, with findings about the impact of the renewal on other stakeholder groups reported separately [13, 14].

### Ethical considerations

This study received ethics approval from the University of Melbourne Human Research Ethics Committee (HREC Reference: 1,852,257). All participants provided written informed consent before participating in the interview.

## Results

Among 87 stakeholders invited to participate in STORIES via direct email, 49 provided informed consent (58%; two emails undeliverable). Interviews were conducted between November 2018 and August 2019, 11 to 20 months after the program transition, and averaged 41 minutes in length (range: 20–69 min).

A total of 24 individuals, representing six stakeholder groups, who spoke about the impact of the Renewal on the pathology sector were included in this analysis (Table 1). Ten pathology sector representatives were interviewed including laboratory directors, pathologists, managers, and staff. Responses from six healthcare providers, and five policy and program staff were also included alongside three additional responses from advocacy staff, clinical education providers, and researchers which have been grouped to avoid potential identification. Data from the remaining stakeholders invited to participate in STORIES is reported elsewhere [13, 14].

**Table 1:** Total number of STORIES participants and subset included in this study

Stakeholder group	All STORIES participants (n)	STORIES participants in this study (n)
Pathology sector representatives	10	10
Healthcare providers	18	6
Policy and program staff	12	5
Advocacy, education providers, researchers	6	3
Other*	3	0

*The other category includes a medical student, medical intern, and consumer representative

### Themes

Four overarching themes were elicited. These were (a) implementation enablers, (b) implementation challenges, (c) missed opportunities, and (d) implementation improvements.

### Implementation enablers: “When there is evidence out there, one has to take action”

Implementation enablers included a high level of trust in the science for primary HPV screening, the adoption of a quality-based framework allowing pathology laboratories to select the HPV NAT assay used and the benefits of molecular testing.

#### Trust in the evidence-base

Most participants expressed that the decision to transition to primary HPV screening was highly acceptable and appropriate. They felt it was evidence-based, timely, and acknowledged that the test was superior to cytology for screening purposes in the Australian setting, which has high HPV vaccination rates, as primary HPV screening is responsive to the vaccination-induced change in disease rates. As one pathology sector representative said:

*“The numbers of high grade [lesions] are going to diminish because of that. And cytology will not be as effective to screen those [vaccinated] women. In this way, HPV is a more sensitive test.” (Pathology sector, P18)*.

Furthermore, several participants noted that more lesions would be detected at an earlier stage, which would ultimately lead to a reduction in cervical cancer cases:

*“If you get a negative HPV test, your chance of having a CIN lesion within five or six years is greatly reduced as its negative predictive value is about 99%. It’s nowhere near that for cytology. So, we’ll be able to pick up infections that we wouldn’t have picked up [otherwise].” (Pathology sector, P12)*.

#### Opportunities for innovation

A second enabler considered as highly acceptable by participants was the adoption of a quality-based framework allowing laboratories to select their own HPV NAT assay. Several participants discussed the advantages of this which included the autonomy for laboratories to make their own decisions around which HPV NAT assay would suit them. This flexibility is a benefit to the Program as described by one participant:

*“Say, for example, if a particular test platform relatively under calls one of the HPV types, you haven’t got a national issue around that… It gives you redundancy, so if there’s a test failure or something goes wrong, you’ve got other platforms you can use. It’s just a nice model that means you’re not dependent on one test and aren’t locked into long-term contracts with one manufacturer.” (Program staff, P7)*.

Furthermore, some participants discussed the implications of being the first country to use a quality-based requirements framework. As identified by the program staff representative below, this includes greater opportunities for innovation and research into different tests:

*“I think it’s really exciting that Australia chose an open platform for their HPV tests…I think there’s a lot of work to do around quality assurance in that space, and this is a great opportunity to compare different tests, share operating rules, training programs… do some world leading research in this space.” (Program staff, P7)*.

#### Benefits of molecular testing

Other implementation enablers identified by some participants included the greater automation of molecular testing in comparison to cytology, and the consequent decreases in processing times and error margins:

*“It’s a different test, it’s more automated, it’s more efficient and easy for labs…it’s actually far less error margin and resolves some of the quality issues that we see with screeners’ manual screening sometimes.” (Policy staff, P10)*.

Some study participants also reported that diagnostic laboratories benefit from having HPV results with each sample:

*“In our lab, where most of our work is diagnostic [follow-up for high-risk patients], we always get HPV with every result. So, for us, in cytology, that’s great having that feedback on each sample.” (Pathology sector, P1)*.

### Implementation challenges: “You’re basically changing the face of that whole sector”

Many participants reflected on the process of implementation and change management being the most challenging, rather than the Program changes themselves, and acknowledged the task’s enormity. Some considered it too difficult, costly, time-consuming, and felt that more support should have been offered from the Government due to significant changes to laboratory infrastructure, resources and staffing, testing and management pathways and the impact of the delayed functionality of the Register. Despite this, as one pathology representative reflected, the Renewal at the time of the interview was functional:

*“I don’t think anybody, even the most cynical of cynical people would have anticipated how difficult it would have been… Everybody would have expected it better, so I think from that aspect the implementation was poor because it didn’t meet with people’s expectation, but now that we’ve done it for a year and there’s a bit of continuity and we’ve learned on our feet … it has been implemented.” (Pathology sector, P23)*.

#### Laboratory infrastructure, resources, and staffing

Participants described the transition of the workload from primarily cytology-based to molecular-based as having major implications for laboratory infrastructure, resources, and staffing. Workforce planning was identified as a major issue, particularly as the volume of cervical cytology being processed significantly decreased. Many participants noted that laboratories had to make most of their cytologists redundant, which caused anxiety and low morale amongst staff in the lead-up to the Renewal date:

*“There was an impact in the sense that staff were quite anxious, I could sense that. There was anxiety amongst staff, until we knew which way we were heading with regards to gynae[cology] work, with regards to the screening work, or until we figured out how much work would go.” (Pathology sector, P33)*.

The delay of the Renewal start date exacerbated staffing issues. Some laboratories had to employ or train new cytologists to ensure they could continue processing samples for an additional seven months, which caused disruptions, and increased their workload as they had to train new staff with less experience, which also had implications for quality assurance. This is highlighted in the following quote:

“*It got to a point where we were preparing for 1st of May and then I was trying to get enough staff to keep going….So I was trying to employ cytology screeners, but there weren’t that many out there, so I had to employ people with not as much experience, then we train them just so that we can actually get the work done. Because we didn’t have enough people, and towards the end there was a lot of absenteeism… Because they were switched off… And then towards the end of 2017, because we do have quality checks and all that so there were a few people who fell out of the quality check, which means that we had to review the whole year’s work.” (Pathology sector, P18)*.

The predicted impact of the Renewal on laboratory workload in the years after its implementation was also raised. Several participants had concerns about managing staffing and service delivery if test volumes fluctuated due to the longer screening interval. They were aware that the volume of tests being processed would come in waves in the first few years after the Renewal, including a rebound as people returned for the second round of screening after 5 years. The impact of this is described by the following pathology sector representative:

*“Certainly coming into 2020, we’ll have much smaller volumes of cervical screening tests. Most women who are transitioning from two yearly to five yearly will have had a test, and so how the lab, and how the business model that adapts to that much less work, and how we as a lab community deal with workforce implications of further downsizing the lab staff after a massive downsize to start with. Bearing in mind that come year six, we’ve got a scale-up again for the second, somewhat smaller peak, if that makes sense.” (Pathology sector, P12)*.

Many study participants raised concerns about the future of the cytology workforce. They discussed the ongoing importance of gynecological cytology in diagnosing cancers but were concerned about the potential loss of expertise:

*“Even though molecular has taken over, gynae[cological] cytology will always have jobs… Because molecular testing just tells us, “Yes, there is an infection,” It doesn’t tell us that there is a malignancy or not. For that we need cytology. And we need cytologists who can screen.” (Pathology sector, P33)*.

#### Complex testing and management pathways

Managing workflow across two departments- molecular for HPV testing and cytology for LBC triage was also identified as a major challenge. Laboratories had to implement additional processes and protocols that also accounted for the complexity of the new testing and management pathways, as described by a pathology sector representative:

*“So, it’s so complicated. I have one sample coming in, but that sample could be subject to just an HPV test, just an LBC, a definite co- test, or a reflex. It’s got four different pathways in a laboratory, not to mention you stick on any STI test that someone’s requested on that.” (Pathology sector, P23)*.

Confusion from general practitioners around the management pathways and which test to order caused additional workload issues. Several study participations reported that laboratories were called for clarification, with some large laboratories receiving numerous calls a day:

*“We became in essence a hotline call centre for hundreds of calls a day with completely confused GPs [general practitioners] and patients. You know, mostly GPs.” (Pathology sector, P23)*.

Laboratories managed incorrect orders for co-tests for low-grade abnormalities, samples that had been prepared incorrectly, and samples from participants aged under 25 years described as symptomatic for billing purposes:

*“What’s happening is they [GPs] think follow up of low-grade abnormalities requires a co-test, and that is all day everyday we’re receiving requests for co-tests. Some even call it test of cure and the patient has only ever had low grade abnormalities.” (Pathology sector, P38)*.

#### Delayed functionality of the Register

Challenges were further compounded by the delay in the Register, with patient histories not fully integrated in the Register by the program transition date. Participants reported having to access histories through separate jurisdictional registers, which was a manual and time-consuming process. Several participants also highlighted potential implications for patient safety, as pathologists relied on up-to-date information on screening histories including previous abnormalities and treatment, in addition to guidelines, to make appropriate recommendations. Consequently, laboratories were often delayed in sending patients their results.

*“And as I understand it, labs often couldn’t get a full history for a particular woman because they had to try and piece together whatever they could from various registers. In the migration, 20% of women, one in five records, were for women who had their records smattered across jurisdictions, that to me is absolutely huge.” (Program staff, P30)*.

### Missed opportunities: “Of course it has to be done the way the Program specifies”

Participants identified two major opportunities for improving equity in the Program that they believed were missed, including the implementation of self-collection and standardised pathology forms.

#### Early implementation of self-collection

The first missed opportunity related to a new self-collection pathway. Laboratories were required to validate self-collection devices in-house before they could test specimens received from clinicians but only one laboratory was accredited to do this from January 2018. Some participants thought this specific technical requirement was not communicated properly ahead of the transition, resulting in confusion among clinicians who were forwarding samples to their regular laboratory as described in the below quote:

*“There were individual women inconvenienced where the doctor had not knowingly told the patient they could self-collect, then put the swab in a vial of ThinPrep and of course we couldn’t process that nor would the [pathology provider] accept it because of course it has to be done the way the Program specifies.” (Pathology sector, P38)*.

Laboratories received self-collected samples but were not able to process them, leading to concerns about the implications of this on improving screening accessibility.

*“I think that is such a potentially positive aspect of the screening program, self-collection, and yet it’s got barriers at laboratory doing the test level, it’s got barriers on billing level, and on multiple levels there’s a barrier to it.”(Pathology sector, P23)*.

#### Lack of standardised pathology forms

The second missed opportunity identified by some participants was the lack of standardised pathology form fields and mandates to improve data quality and completeness. It was viewed as particularly important to collect Aboriginal and Torres Strait Islander status so that the program performance and equity for different populations could be accurately monitored. One researcher noted that while laboratories are required to report this to the register if they receive it, it is not a mandated field; not all general practitioners include this information on the request form, and not all pathology providers include a field on their form to collect this data:

*“I think it should be mandated for every cervical screening test, that the GP is legally obliged to ensure that they have Aboriginal and Torres Strait Islander status on that pathology form and now obviously now they’ve got that link so that now when the laboratory receives it, they obviously have to [record it] as well. And then that goes to NCSR [Register]. I guess that would be the big difference.” (Researcher, P13)*.

### Feedback on the implementation process: “Should’ve been active engagement right from the get-go”

Study participants discussed how the implementation of the Renewal could have been improved including through early stakeholder engagement, increased financial support and clearer clinical guidelines.

#### Strengthening stakeholder engagement

Early and consistent engagement with the sector from the Government and more representation on implementation and Register committees were identified as ways of ensuring a smoother Renewal process for the pathology sector. This was summarised by one pathology sector representative:

*“There should’ve been active engagement with the pathology sector right from the get-go, and we, for instance, sent endless letters requesting specific things, information, all of which was not forthcoming. So, we had to do a lot of it blind. So really, there should’ve been a proper implementation group that actually did implement things, or help people implement things rather than just being missing in action I suppose.” (Pathology sector, P15)*.

Furthermore, participants thought that communication between the Government and the pathology sector, general practitioners and the community was inadequate and led to confusion about roles and responsibilities. Several laboratories elected to provide their own resources, education sessions and webinars to clinicians:

*“I think the information the government provided to clinicians and the community was substandard. We provided our own material, and we were doing so from the point the decision was made right through until the Program was started.” (Pathology sector, P38)*.

#### Financial support

Several participants discussed the financial impact the Renewal had on their laboratory and the lack of government funding provided. While expenses varied between laboratories, most participants discussed the high costs associated with transitioning to a higher workload of molecular-based tests that required new infrastructure, IT, and equipment including molecular testing platforms and LBC vials:

*“Actually, the biggest impact was on the pathology laboratories who had been spending millions of dollars rearranging their structure for the start of the new program, that actually we were the ones who felt the impact the most and the government had not, at the point of announcement, taken that into consideration at all.” (Pathology sector, P38)*.

One participant also highlighted the high cost of staff redundancies, despite needing to pay fewer staff:

*“The staffing has gone down in price but that is offset by redundancies and things like that that occurred as the shift from the old system to new system.” (Pathology sector, P12)*.

The implementation costs were also discussed in reference to the provision of financial support from the Government. Some participants agreed that the subsidy (locally referred to as the Medicare rebate) to cover the cost of laboratories performing primary HPV screening was adequate but these subsidies did not cover the full extent of the costs associated with the Renewal. This is described by one pathology sector representative:

*“It’s probably one of the first pathology items that actually got funded appropriately, covering all the costs. But that said, it probably didn’t cover the cost of the four years of transition, computer programs needing to be rewritten, laboratories needing to be completely refurbished.” (Pathology sector, P15)*.

#### Clearer clinical guidelines

Finally, participants thought that clearer guidelines should have been provided about how to manage symptomatic patients or those previously treated for high-grade disease, noting that the clinical guidelines were unsuitable for their use, and only appropriate for the screening program:

*“There are no standard guidelines, no clinical management guidelines about how to deal with women in a diagnostic setting. For example, what about women who have been treated for high grade disease?” (Pathology sector, P33)*.

## Discussion

This qualitative study documents the early impacts of the Renewal of the Australian Cervical Screening Program on the pathology sector from the perspectives of key stakeholders. The Renewal was the most significant change in the Program since its introduction in 1991. Our findings identified that while the strength of the evidence base for primary HPV screening was an important implementation enabler, the early impacts of implementation on the pathology sector presented significant disruptions and required multifaceted change. These impacts were described across the other three themes identified in this research. Participants in this study discussed the implementation challenges, including the impact of the change in primary test on the pathology workforce and laboratory workflow, and the simultaneous implementation of a new IT system and national Register. Furthermore, missed opportunities, including the limited availability of self-collection were identified as were implementation improvements, which included the need to support the pathology sector through effective change management, communication that was clear, timely, and transparent, and adequate funding sources.

Australia was one of the first countries to transition from cytology to primary HPV cervical screening as part of its national Program [17]. Participants in this study found this to be broadly acceptable. Our findings are novel, showing that the strength of the evidence base for primary HPV screening played a critical role as an implementation enabler in the pathology sector’s support for the Program. Study participants discussed the appropriateness of using primary HPV screening in Australia as a more sensitive test than cytology in a highly vaccinated population. The appropriateness of the test is supported by extensive modelling showing the greater effectiveness and cost-effectiveness of primary HPV screening compared to a cytology-based screening in the Australian context; [18, 19] and the World Health Organization (WHO) recommendation for primary HPV screening based on evidence of its greater sensitivity in comparison to cytology for the detection of high-grade precancerous disease and stronger negative predictive value [20, 21]. Our study findings corroborate that the strength of the evidence base was an important enabler across the range of key stakeholders involved in the implementation in Australia [13, 14]. A recent systematic review identified 25 high-income countries that have adopted recommendations for primary HPV screening as part of the national program, with the majority planning to transition from cytology [22]. Our findings are of significant relevance to the high-income countries with population-based cervical screening programs using cytology, as the strength of the evidence base may also have significant implications for the perceived acceptability and appropriateness of such transitions.

A major implementation challenge identified by study participants was the fluctuation in the types of pathology laboratory services required, due to a very large increase in HPV test volumes and corresponding reduction in cytology tests. To illustrate this, before the program transition 2.2 million cytology tests were processed annually, [23] but since 2018, only 400,000 cytology tests have been processed per year [24]. Participants discussed the implications of this on the feasibility of the Renewal, due to its impact on the cytology workforce and laboratory workflow. The sector encountered further implementation challenges in having to provide redundancy packages for staff and pathways to non-gynaecological work while simultaneously ensuring adequate resourcing for new IT systems, testing, reporting protocols, and infrastructure. These challenges were further exacerbated by the initial delay in the program transition by the Government. It was evident that these factors had significant cost implications for laboratories. The role of laboratories in managing this transition had been identified prior to the Renewal [6, 7]. However, the participants in this study reported receiving limited additional support to mitigate these impacts. These findings highlight the importance of involving all key stakeholders, including the pathology sector in planning and preparation to ensure adequate resourcing can be provided.

Self-collection addresses many barriers to screening participation among under- and never-screened groups in Australia and internationally, including increased privacy, convenience, and comfort [25–28]. The limited availability of self-collection was viewed as a missed opportunity to promote equitable participation in the Program as soon as the opportunity was available. The delay and limited number of laboratories processing self-collected samples was caused by a regulatory issue as the Therapeutic Goods Administration required each laboratory to validate the collection device that they planned to use for self-collection, as this was not listed as an intended use by test manufacturers [11]. In turn, some laboratories were reluctant to promote self-collection for testing [11]. Participants in our study thought that this caused confusion around the availability and requirements of self-collection among clinicians. Confusion about the eligibility criteria and pathology requirements for self-collection was similarly reported in our team’s interviews with STORIES participants from other disciplines, [13] and in other studies of Australian clinicians [27, 29]. Participants in this study reported that this increased the workload of pathology laboratories who were having to provide clarification and manage the follow-up of self-collected samples that were not able to processed. This highlights the importance of clear and timely communication to all stakeholders in supporting the adoption of self-collection by the health system, which was also identified by other stakeholder groups participating in STORIES [13].

Our findings highlighted key feedback for improving the implementation process, as described in theme four, for the pathology sector. This included the importance of clear and timely communication and transparency from the Government to all stakeholders tasked with implementation of the Renewal. The pathology sector’s workload and expenditure were influenced by components of the Renewal outside of its control, including the delayed implementation of the Register, clinician confusion around the new clinical management guidelines, and regulatory requirements. Stakeholders interviewed felt that there needed to be more representation from and consultation with the pathology sector. In particular, greater planning and preparation, together with more input from the pathology sector, may have helped to mitigate some of these impacts experienced by the sector.

Strengths of this study include its timeliness in reporting on the early impacts on pathology providers in transitioning to primary HPV cervical screening, which is important as an increasing number of countries are also planning to make this change. A broad range of stakeholders were purposively sampled, including ten pathology sector representatives from public and private laboratories across Australia allowing for diverse and relevant perspectives to be obtained. However, a limitation is that these stakeholders may not have represented the views or experiences of all pathology laboratories providing services to the Program. Furthermore, STORIES participants from other stakeholder groups included in this analysis were not prompted to discuss the pathology sector and therefore may have been more likely to comment on challenges for the pathology sector which may have been more salient than implementation enablers. Another strength was that the interview guide was developed using Proctor’s outcomes for implementation research, a widely used implementation framework [15]. This ensured the breadth of experiences was captured to provide an indication of the success of the early implementation outcomes [15]. Illustrating this, many participants discussed the implementation costs associated with the Renewal, which is an implementation outcome that is not widely reported in implementation studies, including those on self-collection for cervical screening [30–32]. Future research should consider the impact of implementation costs on the acceptability of the transition. As the Program is now in its sixth year, research assessing medium and longer-term implementation outcomes such as penetration and sustainability will provide important insights into how many of the early impacts have been resolved. Research currently being conducted by our team with the pathology sector will allow us to assess these implementation outcomes.

Our qualitative study about the early impacts of Australia’s decision to transition to primary HPV cervical screening on pathology services highlighted the most significant implementation enablers, barriers, missed opportunities and possible improvements. The change to primary HPV screening, alongside new IT systems and a Register, had a significant impact on the Australian pathology sector. Laboratories, tasked with the implementation of the Renewal, faced significant workflow, workload, staffing, infrastructure, and financial challenges. These findings can help countries transitioning to primary HPV screening at all stages of the implementation process anticipate barriers and implement strategies to support the transition. We identified the importance of effective change management, early and active engagement with the sector, adequate funding, and transparent, clear and timely communication as enablers to change that cannot be underestimated in supporting the pathology sector to implement primary HPV cervical screening.

### Electronic supplementary material

Below is the link to the electronic supplementary material.

Supplementary Material 1

## Data Availability

The data generated and/or analysed in this study are not publicly available to protect the privacy and confidentiality of individual participants. Data may be available from the corresponding author on reasonable request if appropriate ethics approvals are in place. All authors had full access to the data for this study.

## List of abbreviations

HPV: Human papillomavirus
IT: Information technology
LBC: Liquid-based cytology
NAT: Nucleic acid test
STORIES: STakeholder Opinions of Renewal Implementation and Experiences Study
