# Supplementary material for: The early impacts of primary HPV cervical screening implementation in Australia on the pathology sector: a qualitative study
Source: BMC Health Serv Res. 2023 Oct 6;23:1073. doi: 10.1186/s12913-023-10040-6 (PMC10559573; doi:10.1186/s12913-023-10040-6)
Supplement: Supplementary file 1 — Supplementary Material 1 [file 12913_2023_10040_MOESM1_ESM.docx]

**COREQ Checklist**

**The early impacts of primary HPV cervical screening implementation in Australia on the pathology sector: a qualitative study**

| **Item number** | **Guide questions/description** | **Reported on Page #** | **Comment** |
| --- | --- | --- | --- |
| **Domain 1: Research team and reﬂexivity** | | | |
| *Personal Characteristics* | | | |
| 1. Interviewer/facilitator | Which author/s conducted the interview or focus group? | 7 | - Julia ML Brotherton - Megan A Smith - Tracey McDermott - Nicole M Rankin - Farhana Sultana - Dorothy A Machalek |
| 2. Credentials | What were the researcher’s credentials? e.g. PhD, MD | N/A | All authors have a bachelor’s degree, masters degree and/or a PhD. |
| 3. Occupation | What was their occupation at the time of the study? | 8 | All authors were employed in a research capacity at the time of the study across various research institutes/organisations. |
| 4. Gender | Was the researcher male or female? | N.A. | All authors are female. |
| 5. Experience and training | What experience or training did the researcher have? | 7, 8 | All authors have received university-level training in qualitative data collection and analysis. |
| *Relationship with participants* | | | |
| 6. Relationship established | Was a relationship established prior to study commencement? | N/A. | No relationship was established specifically for the purpose of this study. As most interviewers have been involved in cervical screening research for some time, some participants were known to several authors and potentially to their interviewer. |
| 7. Participant knowledge of the interviewer | What did the participants know about the researcher? e.g. personal goals, reasons for doing the research | N/A. | Participants received a plain language statement informing them about the study and the research team. |
| 8. Interviewer characteristics | What characteristics were reported about the interviewer/facilitator? e.g. Bias, assumptions, reasons and interests in the research topic | N/A. | None. |

| **Domain 2: study design** | | | |
| --- | --- | --- | --- |
| *Theoretical framework* | | | |
| 9. Methodological orientation and Theory | What methodological orientation was stated to underpin the study? e.g. grounded theory, discourse analysis, ethnography, phenomenology, content analysis | 8 | Inductive thematic analysis |
| *Participant selection* | | |  |
| 10. Sampling | How were participants selected? e.g. purposive, convenience, consecutive, snowball | 7 | Purposive recruitment method |
| 11. Method of approach | How were participants approached? e.g. face-to-face, telephone, mail, email | 7 | Email |
| 12. Sample size | How many participants were in the study? | 9 | There were 49 participants in STORIES. This study reported on a subset of this data from 24 participants. |
| 13. Non-participation | How many people refused to participate or dropped out? Reasons? | 9 | The response rate was 58%. The reasons for not participating are unknown. Two emails were undeliverable. |
| *Setting* | | | |
| 14. Setting of data collection | Where was the data collected? e.g. home, clinic, workplace | 7 | Data was collected online, by phone or face-to-face in the workplace of the interviewer or interviewee. |
| 15. Presence of non-participants | Was anyone else present besides the participants and researchers? | N/A. | No one else was present for the interview. |
| 16. Description of sample | What are the important characteristics of the sample? e.g. demographic data, date | 9 | The primary paid role of the participants is reported. |
| *Data collection* | | | |
| 17. Interview guide | Were questions, prompts, guides provided by the authors? Was it pilot tested? | 7-8 | The types of questions included in the interview are provided. The interview guide was not piloted tested, but it was reviewed by an Advisory Committee of Australian subject matter experts. |
| 18. Repeat interviews | Were repeat interviews carried out? If yes, how many? | N/A. | Repeat interviews were not conducted. |
| 19. Audio/visual recording | Did the research use audio or visual recording to collect the data? | 7 | Interviews were audio recorded. |
| 20. Field notes | Were ﬁeld notes made during and/or after the interview or focus group? | N/A. | No |
| 21. Duration | What was the duration of the interviews or focus group? | 9 | Interviews ranged in time from 20-69 minutes. |
| 22. Data saturation | Was data saturation discussed? | N/A. | Purposive sampling was used to obtain a diverse sample of stakeholders from across Australia involved in the implementation of the Renewal rather than to obtain data saturation. |
| 23. Transcripts returned | Were transcripts returned to participants for comment and/or correction? | 7 | Participants were asked if they would like to review a copy of their transcript to review it for accuracy and completeness. |
| **Domain 3: Analysis and ﬁndings** | | | |
| *Data analysis* | | | |
| 24. Number of data coders | How many data coders coded the data? | 8 | For this STORIES paper, three authors coded the data:   - Claire Bavor - Khic Houy-Prang - Tracey McDermott   All other authors were involved in the discussion of the coding tree and themes. |
| 25. Description of the coding tree | Did authors provide a description of the coding tree? | 8 | A coding tree to identify the key themes across all the transcripts included in STORIES was developed. All data that had been coded as relating to the pathology sector underwent a second round of coding to identify themes specific to the impact of the Renewal on the pathology sector. |
| 26. Derivation of themes | Were themes identiﬁed in advance or derived from the data? | 8 | Themes were derived from the data using inductive thematic analysis. |
| 27. Software | What software, if applicable, was used to manage the data? | 8 | NVivo 11 and NVivo 12 |
| 28. Participant checking | Did participants provide feedback on the ﬁndings? | N/A. | Participants did not provide feedback. |
| *Reporting* | | | |
| 29. Quotations presented | Were participant quotations presented to illustrate the themes/ﬁndings? Was each quotation identiﬁed? e.g. participant number | 10-20 | Participant quotes are included to illustrate each of the findings, alongside their role and participant number. |
| 30. Data and ﬁndings consistent | Was there consistency between the data presented and the ﬁndings? | 10- | There was consistency between the data and findings. |
| 31. Clarity of major themes | Were major themes clearly presented in the ﬁndings? | 10-20 | Four overarching themes have been presented. Subthemes have been included. |
| 32. Clarity of minor themes | Is there a description of diverse cases or discussion of minor themes? | 10-20 | Minor themes have been included in the results section. For example, lack of a standardised pathology form is a minor theme that has been discussed. |
